# gga-miRNA-18b-3p Inhibits Intramuscular Adipocytes Differentiation in Chicken by Targeting the *ACOT13* Gene

**DOI:** 10.3390/cells8060556

**Published:** 2019-06-07

**Authors:** Guirong Sun, Fang Li, Xiangfei Ma, Junwei Sun, Ruirui Jiang, Yadong Tian, Ruili Han, Guoxi Li, Yanbin Wang, Zhuanjian Li, Xiangtao Kang, Wenting Li

**Affiliations:** College of Animal Science and Veterinary Medicine, Henan Agricultural University, Zhengzhou 450002, China; grsun@henau.edu.cn (G.S.); fangli0909@126.com (F.L.); mxf1228@126.com (X.M.); jw2013au@126.com (J.S.); jrrcaas@163.com (R.J.); ydtian111@163.com (Y.T.); Rlhan@126.com (R.H.); liguoxi0914@126.com (G.L.); ybwang2008@126.com (Y.W.); lizhuanjian@163.com (Z.L.); xtkang2001@263.net (X.K.)

**Keywords:** miR-18b-3p, *ACOT13*, intramuscular adipocytes, differentiation

## Abstract

Intramuscular fat (IMF) is the most important evaluating indicator of chicken meat quality, the content of which is positively correlated with tenderness, flavor, and succulence of the meat. Chicken IMF deposition process is regulated by many factors, including genetic, nutrition, and environment. Although large number of omics’ studies focused on the IMF deposition process, the molecular mechanism of chicken IMF deposition is still poorly understood. In order to study the role of miRNAs in chicken intramuscular adipogenesis, the intramuscular adipocyte differentiation model (IMF-preadipocytes and IMF-adipocytes) was established and subject to miRNA-Seq. A total of 117 differentially expressed miRNAs between two groups were obtained. Target genes prediction and functional enrichment analysis revealed that eight pathways involved in lipid metabolism related processes, such as fatty acid metabolism and fatty acid elongation. Meanwhile a putative miRNA, gga-miR-18b-3p, was identified be served a function in the intramuscular adipocyte differentiation. Luciferase assay suggested that the gga-miR-18b-3p targeted to the 3′UTR of *ACOT13*. Subsequent functional experiments demonstrated that gga-miR-18b-3p acted as an inhibitor of intramuscular adipocyte differentiation by targeting *ACOT13*. Our findings laid a new theoretical foundation for the study of lipid metabolism, and also provided a potential target to improve the meat quality in the poultry industry.

## 1. Introduction

Meat products are essential for humans to maintain a balanced diet. Recently, great progress has been achieved in meat quantity by genetic selection for growth rate and meat yield. However, the strong selection led to the decline in the meat quality, which presented as larger fiber diameters, higher proportion of glycolytic fibers, and lower intramuscular fat (IMF) [1,2]. Among them, IMF was considered to be the main factor affecting meat quality. It has been demonstrated that IMF content, the amount of fat within muscles, was positively correlated with flavor, juiciness, and tenderness [3]. Gushi chicken as a Chinese local chicken breed with high meat quality, has been favored by most consumers for its delicious and unique-flavored meat. Numerous studies have shown that intramuscular fat not only correlated with muscle flavor, but also related to many other meat quality traits (such as muscle pH, hydraulics, tenderness, etc.) [2,3,4,5,6]. Our previous studies have found that there were significant differences in the density and size of lipid droplets in the breast muscle between the juvenile and late-laying period of Gushi hens. As expected, significant difference in meat quality was also captured between such groups. That is the higher the meat quality the late-laying period group showed, the more lipid droplets were in their breast muscles [4].

Intramuscular fat mainly consisted of intramuscular adipocytes, which were differentiated by intramuscular preadipocytes. In recent years, many previous studies focused on intramuscular adipocytes in various species, such as cattle and pig [5,6]. Seldom research performed on chicken. This study tried to elucidate the mechanism of chicken IMF deposition by establishing the intramuscular adipocyte differentiation model. 

MicroRNA (miRNA) is a class of endogenous noncoding single-stranded RNA, with 18 to 25 nucleotides (nt) in length, which commonly acted as the negative regulators of gene expression at post-transcriptional levels [7]. miRNAs played important roles in regulating a large number of biological and metabolic processes [8], including cell growth, proliferation, differentiation, and cell death. It has been reported that miRNAs regulated the biological functions of adipocyte, such as adipogenic differentiation and white adipocyte browning [9,10]. Several miRNAs have been identified to be responsible for adipocyte differentiation or adipogenesis [11,12]. Furthermore, published data showed that miR-18b could promote food intake, induce the expansion of adipose tissues and modulate fatty acid content, utilization and oxidation [13,14]. Sex hormones were essential for regulating adipocyte metabolism and affecting specific fat store [15,16]. In human, fat distribution was regulated by the concentration of sex hormones [17]. Estrogen almost regulated the transcription of important genes in various processes such as fat synthesis, fat transportation and oxidation hydrolysis [18,19,20]. Lack of estrogen could lead to obesity directly [21]. Cooke et al. have reported that estrogen determined the number of adipocytes by affecting food intake and energy consumption, thereby inhibited the fat deposition in the body [22]. Jinhui-wu et al. found that miR-18b played a negative regulatory role in human retinal endothelial cells (HRECs) proliferation by targeting insulin growth factor-1 (IGF-1). High glucose inhibited the expression of miR-18b and then the inhibitory effect of miR-18b resulted in an increasing expression of IGF-1 gene [23]. IGF-1 receptor was located in the 3T3-L1 preadipocyte and adipocyte, and IGF-1 receptor signaling played a key role in inducing the 3T3-L1 preadipocytes differentiating into adipocytes [24,25]. It has been demonstrated that a high level of insulin or IGF-1 could lead to the initiation of the 3T3-L1 preadipocyte differentiation, which finally induced the cell differentiation and affected fat production [26]. In conclusion, we inferred that miR-18b played an important role in the differentiation of adipocytes.

The aim of this study was to identify the miRNAs affecting IMF deposition between IMF-preadipocytes and IMF-adipocytes by using small RNA deep sequencing. Differential expressing miRNAs and their target genes were identified to elucidate the regulatory patterns of miRNAs and their network. According to the analysis, a candidate miRNA, gga-miR-18b-3p, was revealed to be potentially associated with IMF deposition. Subsequently, further validation for miR-18b-3p has been performed. Our results may contribute to a better understanding of chicken IMF deposition, and also provide a target for subsequent investigation to improve meat quality in the poultry industry.

## 2. Materials and Methods

### 2.1. Ethics Statement

All the procedures of animal experimentation in this study strictly followed the protocol approved by the Institutional Animal Care and Use Committee (IACUC) of Henan Agricultural University (Permit Number: 17-0118).

### 2.2. Cell Isolation, Culture, and Differentiation of Primary Preadipocytes

Primary chicken intramuscular preadipocytes were isolated from breast muscle tissue of two-week-old chickens following the methods as described previously [4]. Then cells were seeded in 6-well plates and maintained with complete medium (DMEM/F12 (1:1), 10% FBS, and 1% penicillin/streptomycin) as described by Ding et al. (2015). Once upon the confluence of cells reaching 90%, the medium would be replaced with differentiation medium (0.5 mM 3-isobutyl-1-methylxanthine, 1 μM dexamethasone, 10 g/l insulin, and 1 μM oleic acid) for inducing the cell differentiation. Undifferentiated cells were named as IMF-preadipocytes, while cells collected at 10 days post-induction were named as IMF-adipocytes.

### 2.3. Oil Red O Staining and Cellular TG Content Measurement 

Cells were fixed for 30 min with 4% paraformaldehyde, incubated for 20 min at room temperature with Oil Red O, and then visualized with light microscopy following the methods published [4]. TG content was measured in a triglyceride content detection kit (APPLYGEN, Beijing, China) according to the manufacturer’s instructions.

### 2.4. Small RNAs Library Construction and Deep Sequencing

Total RNA of IMF-preadipocytes and IMF-adipocytes were prepared using mirVana™ miRNA Isolation Kit (Austin TX, US). After quality checking, ~2 μg total RNA for each sample was used to construct the small RNA library according to the protocol (Illumina Small RNA Sample Prep Kit). A total of 4 libraries (IMF-preadipocytes and IMF-adipocytes, each with 2 replicates) were sequenced with Illumina Genome Analyzer (Illumina, San Diego, CA, USA). Meanwhile, cDNA was also prepared using PrimeScript™ RT reagent kit with gDNA Eraser (Takara, Dalian, China) and stored at −20 °C for the following wet-lab experiment.

### 2.5. Identification and Prediction of miRNAs

After a series of quality control, clean reads for each sample were mapped to Gallus gallus 5.0 using Bowtie (http://bowtie.cbcb.umd.edu) (The University of Maryland, Maryland, MD, America) [27], and then aligned with the known miRNAs in miRbase [28] with allowing less than one mismatch to identify known miRNAs. The identified miRNAs were classified into families based on their sequence similarities. Unmatched reads remained were further processed to predict novel miRNAs using miRCat [29]. RegRNA 2.0 was applied to predict the structures of novel miRNAs precursors, hairpins, secondary structures, and minimum free energy [30].

### 2.6. Prediction of miRNA Target Genes and GO and Pathway Enrichment Analysis

Differentially expressed miRNAs with *p* value ≤ 0.05 were identified and their target genes were predicted by TargetScan (http://www.targetscan.org/) and miRanda (microRNA.org). Correlation networks was visualized using Cytoscape software [31]. Gene ontology (GO) enrichment and Kyoto Encyclopedia of Genes and Genomes (KEGG) analysis were performed to investigate the underlying functions and some vital pathways involved in lipid metabolism using DAVID (The Database for Annotation, Visualization, and Integrated Discovery) database (https://david.ncifcrf.gov/). 

### 2.7. Functional Assays

#### 2.7.1. Vector Construction 

The chicken *ACOT13* coding region was cloned using primer ACOT13(CDS), isolated with EcolΙ and HindIII, then inserted into pcDNA3.1-EGFP to obtain the pcDNA3.1-ACOT13. Similarly, a fragment amplified from the GFP gene was also cloned into pcDNA3.1-EGFP as a negative control (NC). The 3′ UTR region of *ACOT13* gene fragments containing the predicted target site, including wild type (WT) and mutant type (MUT), were cloned into psiCHECK2 vector (isolated using XhoΙ and NotΙ (Takara, Dalian, China)). The recombinant vectors were named as ACOT13-3′UTR-WT and ACOT13-3′UTR-MUT, respectively. The primers are shown in Table 1. 

#### 2.7.2. Luciferase Assays 

DF1 cell, a cell line of chicken embryo fibroblasts, was used to validate the miRNA target. Cells were seeded into 24-well plates. Co-transfection with 500 ng ACOT13-3′UTR-WT or ACOT13-3′UTR-MUT and 2 μL of gga-miR-18b-3p mimics or negative control were performed with Lipofectamine 2000 (Invitrogen). Then luciferase activities were measured using the Dual Luciferase Reporter Assay System (Promega, WI, USA) at 48 hours post-transfection. Assays were performed in triplicate.

#### 2.7.3. Overexpression and Knockdown Assay in Preadipocytes 

Primary preadipocytes were seeded into 6-well plates and transfected with gga-miR-18b-3p mimics or si-ACOT13 (small interfering RNA of *ACOT13* gene, GenePharma, Shanghai, China), meanwhile their non-targeting sequences were used as negative control. Furthermore, a rescue experiment was conducted after *ACOT13* gene knockdown in preadipocytes, that was to transfect the pcDNA3.1-ACOT13 into cells. Post-transfected cells were collected for analyzing the transfection efficiency, the cell differentiation, and gene expression at 48 hours post-transfection.

#### 2.7.4. Quantitative PCR

cDNA was obtained as mentioned above in Section 2.4. Specially, the cDNA synthesis and quantitative PCR (qPCR) for miRNAs were performed with Bulge-loopTM miRNA qPCR Starter Kit (Ribobio, Guangzhou, China). β-actin and U6 genes were used as reference genes for genes and miRNAs respectively. qPCR amplification was conducted as described previously [32,33]. All the primers used are listed in Table 1. 

### 2.8. Statistical Analysis

Relative gene expression obtained from q-PCR data was calculated using the 2^−ΔΔCt^ method [34]. All of the data was presented as the mean ±SE (standard error) with at least three independent replicates and visualized using the “ggplot2” package in R (version 3.2.2, the University of Auckland, Auckland, New Zealand) and GraphPad Prism 7 software (San Diego, CA, USA). Significant differences in the data between groups were tested by Student’s t-test at 5% level using SPSS 19.0 software (IBM, Chicago, IL, USA).

## 3. Results

### 3.1. Phenotypic Variations between IMF-Preadipocytes and IMF-Adipocytes

**Figure 1:**
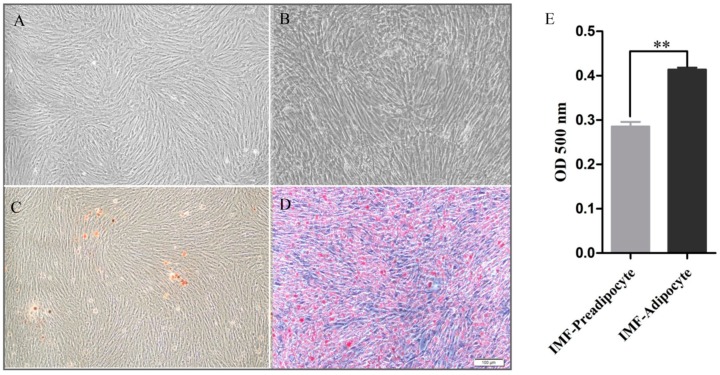
Introduction of intramuscular preadipocytes in vitro. (**A**) IMF (intramuscular fat)-preadipocytes. (**B**) Oil Red O staining of IMF-preadipocytes. (**C**) IMF-adipocytes. (**D**) Oil Red O staining of IMF-adipocytes. (**E**) Triacylglycerol (TG) content of IMF-preadipocytes and IMF-adipocytes group. Data is shown as mean ± SE. ** *p* < 0.01.

To visualize the difference of adipogenesis between IMF-preadipocytes and IMF-adipocytes, the morphological characteristics of chicken IMF-preadipocytes and IMF-adipocytes are shown in Figure 1. At 0 day, IMF-preadipocytes we captured were shown in presence (Figure 1A) or absence of Oil Red O staining (Figure 1B). At 10 days after differentiation induction, IMF-preadipocytes were fully differentiated into IMF-adipocytes filled with lots of lipid droplets (Figure 1C,D). Triacylglycerol content in the IMF-adipocytes was significantly higher than that in IMF-preadipocytes (Figure 1E).

### 3.2. Summary of Sequencing Data

**Figure 2:**
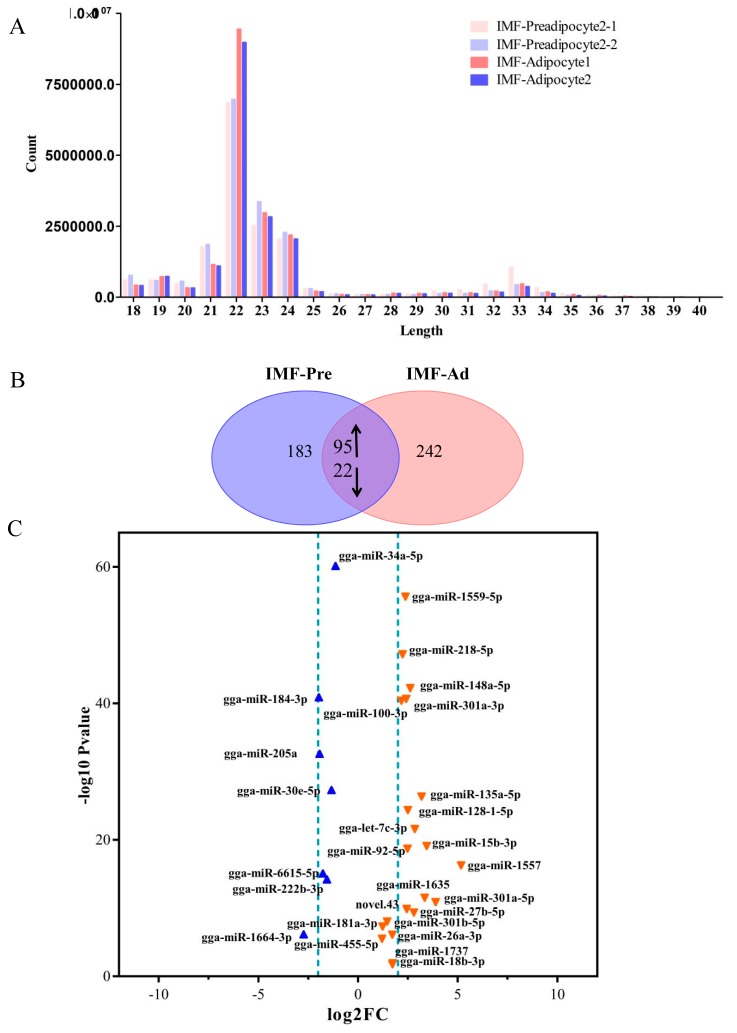

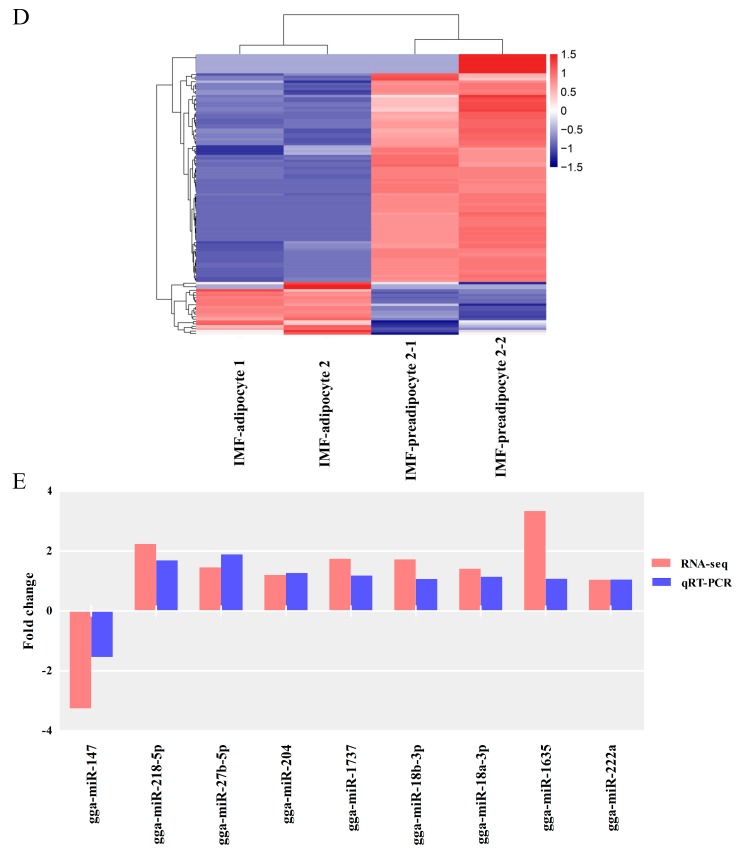
Small RNA sequencing and analysis of differentially expressed miRNAs. (**A**) Distribution of the length and abundance of small RNA sequences in chicken adipocytes. (**B**) Differential expression of miRNA in IMF-preadipocytes overlapping with that in IMF-adipocytes. (**C**,**D**) Volcano plot and heatmap of differentially expressed miRNAs between preadipocytes and adipocytes. (**E**) Verification of sequencing results by qPCR.

Four small RNA libraries were constructed as follows: IMF-Preadipocyte 2-1, IMF-Preadipocyte 2-2, IMF-Adipocyte 1, and IMF-Adipocyte 2, with two replicates for each treatment. And then were sequenced separately using an Illumina Genome Analyzer System. After discarding low-quality reads and masking adaptor sequences, clean reads with 21 to 24 nt in length were obtained, with the vast majority being 22 nt in length, which was consistent with the common size of miRNAs (Figure 2A). After quality control and adaptor removal, a total of 19,178,226; 19,786,345; 20,109,174; and 19,530,973 clean reads were obtained from four libraries respectively. The reads count of each library were about 20 M with more than 90% of Q20 score. The percentage of the 22 nt reads of the total were 37.68%, 38%, 48.83% and 49.56% for the four libraries. The high-quality reads were subsequently annotated to different categories of RNA (identified miRbase, repeats-associated RNA, rRNA, tRNA, snRNA, snoRNA, etc.) using databases such as miRBase (V19.0) [35] and Genbank (Gallus_gallus-5.0). The most abundant RNA species (based on read count) in the four libraries was classified as miRNAs, accounting for 71.58%, 56.53%, 72.41%, and 57.06% in the four libraries. This indicated that the deep sequencing data were highly enriched for mature miRNA sequences and well suitable for subsequent profiling analysis. The second most abundant category was rRNAs, accounting for 4.36%, 8.34%, 3.89%, and 14.02% in the four libraries, respectively. In addition, unknown RNAs also represented a high percentage (17.82%, 29.08%, 17.65%, and 24.13%, respectively). Finally, all reads were aligned against the chicken genome using the program miRCat [34]. 

### 3.3. Screening for Differentially Expressed miRNAs 

To identify the major miRNAs involved in intramuscular fat deposition in chicken, differentially expressed miRNAs were screened between the IMF-preadipocytes and IMF-adipocytes groups. In total, 117 differential miRNAs were identified, including 91 known miRNAs and 26 novel miRNAs (Table S1). Of these, 95 were up-regulated and 22 were down-regulated miRNAs in IMF-adipocytes relative to IMF-preadipocytes (Figure 2B,C). Furthermore, clustering analysis indicated that high similarity was shown within two replicates for each group (Figure 2D). To double check the results of miRNA-seq, nine differentially expressed miRNAs, including eight down-regulated and one up-regulated, were randomly selected for qPCR assay. Figure 2E showed the result of relative expression level from qPCR, which was consistent with the miRNA-Seq data. It indicated that identification and abundance estimation of miRNAs were reliable in this study.

### 3.4. Target Gene Prediction, Functional Enrichment Analysis, and Candidate Genes Identification

**Figure 3:**
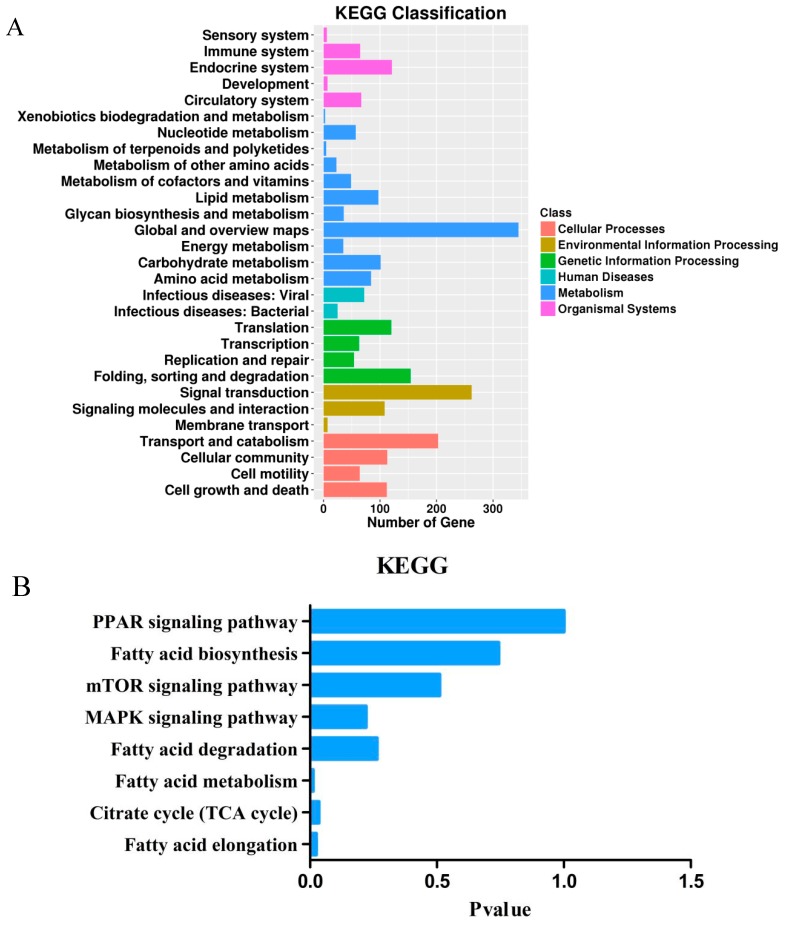

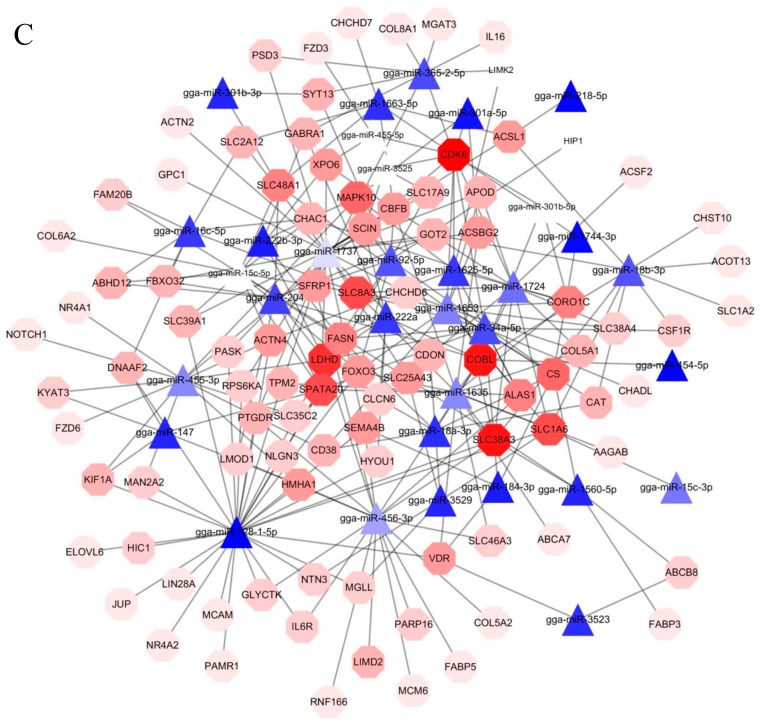
Functional enrichment analysis of target genes. (**A**) KEGG (Kyoto Encyclopedia of Genes and Genomes) analysis on targets of differentially expressed miRNAs. (**B**) Identified KEGG pathways related to lipid metabolism. (**C**) Interactive network for differentially expressed miRNAs and their targets.

To better understand the potential miRNAs responsible for adipogenesis in chicken, target prediction of differentially expressed miRNAs and functional enrichment analysis were conducted. A total of 4005 target genes were identified by 117 differentially expressed miRNAs. All target genes were subjected to enrichment analysis of functions and signaling pathways. Biological processes significantly enriched in adipogenesis-related terms, including regulation of cell differentiation (GO:0045595), cellular lipid metabolic process (GO:0044255) and lipid metabolic process (GO:0006629) (Table S2). Furthermore, many lipid-related pathways were also enriched by target genes, such as fatty acid metabolism (gga01212), fatty acid elongation (gga00062), citrate cycle (TCA cycle) (gga00020) and fatty acid degradation (gga00071) (Figure 3A). Eight pathways related to lipid metabolism were focuse (Figure 3B and Table 2). Of these, “fatty acid elongation” was significantly enriched by the *ELOVL* gene family, *HACD* gene family, *ACOT7,* etc. Lots of published data has proven that the *ELOVL* and *HACD* gene families catalyzed the reactions of the long-chain fatty acids elongation cycle [35,36,37]. Additionally, it has already been reported that the *ACOT* gene family, such as *ACOT7* and *ACOT13*, played a key role in the regulation of hepatic lipid metabolism [38,39]. 

In order to clarify the roles of miRNAs and genes in regulating lipid metabolism, a network was established with Cytoscape. Among these differentially expressed miRNAs, miR-18b-3p was noticed with its targets, the *ACOT13* gene (Figure 3C). It was inferred that gga-miR-18b-3p might be an important miRNA responsible for lipid metabolism.

### 3.5. Expression of gga-miR-18b-3p and ACOT13 in Preadipocytes and Adipocytes

**Figure 4:**
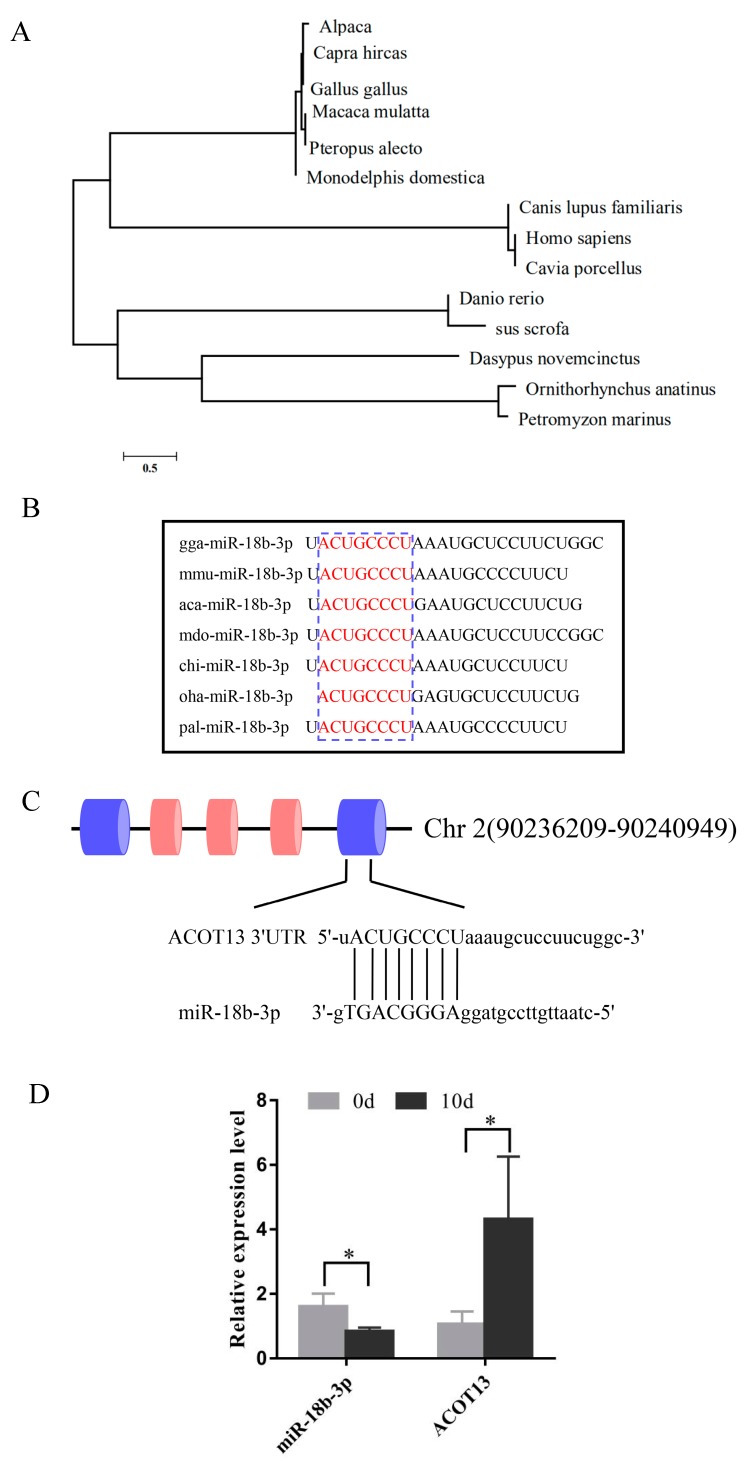
The *ACOT13* gene was a potential target gene of miR-18-3p. (**A**) Cluster analysis showed miR-18b-3p was conserved in various species. (**B**) Conservatism analysis of the miR-18b-3p seed region. (**C**) The interaction of miR-18b-3p and *ACOT13* was predicted based on miRCat. (**D**) Expression of miR-18b-3p and *ACOT13* in pre-differentiation (0 day) and post-differentiation (10 days).

To illustrate the homology of miR-18b-3p in various species, cluster analysis was performed, which indicated that chicken miR-18b-3p was highly identical with *alpaca* and *Capra hircas* (Figure 4A). Besides, the seed region of the mature miR-18b-3p sequence was highly conserved in mouse, reptile, chicken, goat, etc. (Figure 4B). A potential binding site for miR-18b-3p was identified in the 3′UTR region of the *ACOT13* gene (Figure 4C). Furthermore, the expression pattern of gga-miR-18b-3p and its putative target *ACOT13* were detected between pre-differentiation (0 day) and post-differentiation (10 days). As shown in Figure 4D, oppose expression pattern of miR-18b-3p and *ACOT13* gene was observed at 10 days post-differentiation (Figure 4D). It indicated that there was a negative correlation between the expression of miR-18b-3p and the *ACOT13* gene.

### 3.6. miR-18b-3p Functioned as an Inhibitor during Preadipocytes Differentiation by Targeting the ACOT13 Gene

**Figure 5:**
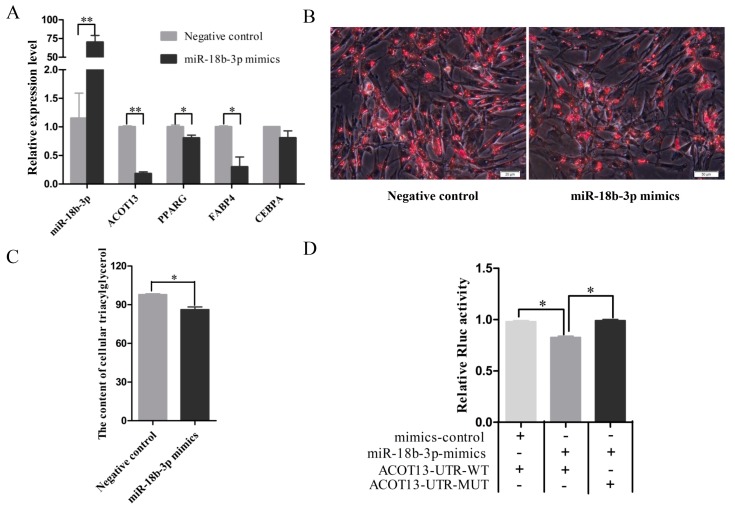
miR-18b-3p inhibited adipocyte differentiation by targeting *ACOT13*. (**A**) The expression level of miR-18b-3p and marker genes of adipocyte differentiation after miR-18b-3p overexpression. (**B**) Number of lipid droplets with miR-18b-3p overexpression. (**C**) The concentration of intracellular triglyceride in the miR-18b-3p overexpressing group was significantly lower. (**D**) Verification of miR-18b-3p target gene using luciferase assay.

To investigate the regulatory role of gga-miR-18b-3p on preadipocyte differentiation, an over-expression experiment was conducted by transfecting gga-miR-18b-3p mimics into chicken preadipocytes. Compared with negative control, the expression level of gga-miR-18b-3p was significantly increasing in the overexpression group (Figure 5A). Expectedly, the expression levels of *ACOT13*, *PPARG*, and *FABP4* were significantly decreased in the gga-miR-18b-3p overexpressing group relative to negative control. The differentiation of preadipocytes was determined using Oil Red O staining and intracellular triglycerides concentration. Evidence from Oil Red O staining suggested that the number of lipid droplets was decreased in the overexpression group compared with the control group (Figure 5B). Furthermore, the concentration of intracellular triglyceride in the gga-miR-18b-3p overexpressing group was significantly lower than that of the negative control group (Figure 5C). All of the above results demonstrated that gga-miR-18b-3p functioned as an inhibitor for preadipocytes differentiation. 

In order to confirm the gga-miR-18b-3p target on *ACOT13*, a plasmid-containing wild-type or mutant target site of the 3′UTR region of the *ACOT13* gene was transfected into DF1 cells together with gga-miR-18b-3p or negative control mimics. gga-miR-18b-3p mimics significantly decreased the luciferase activity of ACOT13-3′UTR-WT, but it did not affect the activity of ACOT13-3′UTR-MUT (Figure 5D). This result indicated that gga-miR-18b-3p could directly bind on the 3′UTR region of the *ACOT13* gene. Taken together, these results suggested that miR-18b-3p inhibited the preadipocytes differentiation by down-regulating the *ACOT13* gene. 

### 3.7. ACOT13 Promoted Preadipocyte Differentiation by Fatty Acid β-Oxidation and Lipogenic Transcription

To elucidate the function of *ACOT13* on chicken preadipocyte differentiation, overexpression, knockdown and rescue experiment of the *ACOT13* gene in chicken intramuscular preadipocytes were designed. After transfection for 48 hours, expression of marker genes that involved in fatty acid β-oxidation and adipogenesis were investigated. Meanwhile, the content of intracellular triglyceride was measured. 

**Figure 6:**
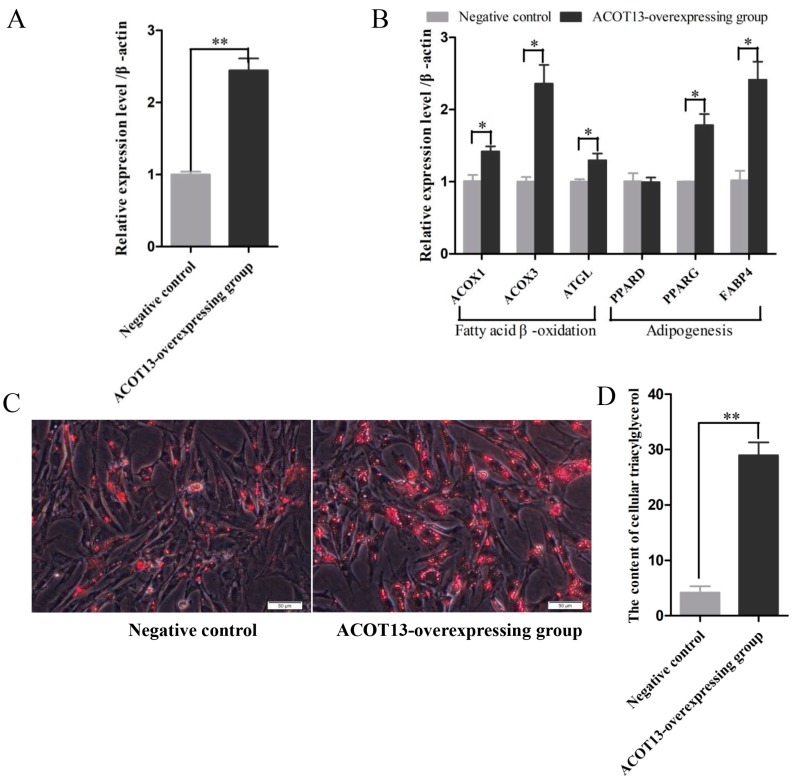
Overexpression of *ACOT13* promoted adipocyte differentiation in intramuscular adipocytes. (**A**) *ACOT13* expression level was induced in the ACOT13 overexpressing group. (**B**) Levels of *ACOX1*, *ACOX3*, *ATGL*, *PPARG,* and *FABP4* mRNAs increased after *ACOT13* overexpression. (**C**) Overexpression of the *ACOT13* gene increased the lipid droplet formation in intramuscular adipocytes by Oil Red O staining. (**D**) In the ACOT13 overexpressing group, triacylglycerol synthesis was increased. * *p* < 0.05; ** *p* < 0.01.

First, overexpression of the *ACOT13* gene in chicken intramuscular preadipocytes. Compared with the negative control group, the expression level of *ACOT13* significantly increased (Figure 6A). The marker genes related to fatty acid β-oxidation, including *ACOX1*, *ACOX3,* and *ATGL*, were significantly higher in the ACOT13 overexpressing group relative to the control group (Figure 6B). Meanwhile, marker genes involved in adipogenesis, including *PPARG* and *FABP4*, also presented a higher expression level in the ACOT13 overexpressing group relative to the control (Figure 6B). Results from Oil Red O staining and content of intracellular triglyceride suggested that the accumulation of lipid droplets significantly increased in the ACOT13 overexpressing group compared with the control (Figure 6C,D). 

**Figure 7:**
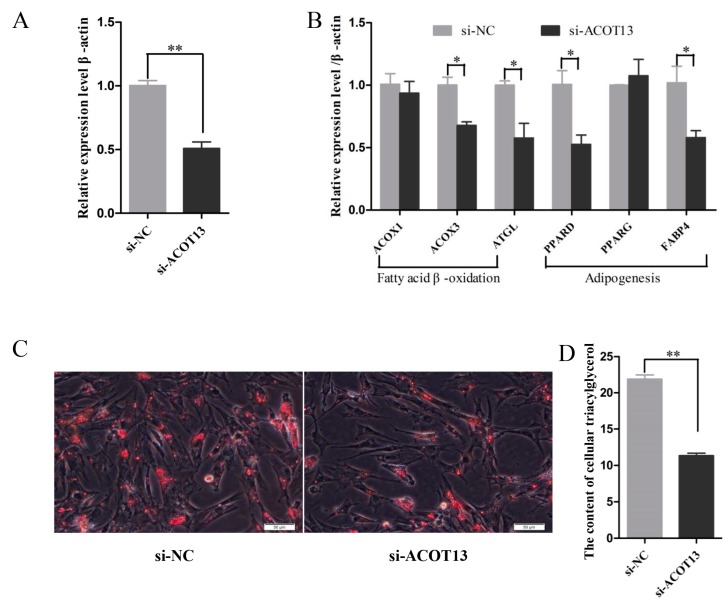
Knockdown of *ACOT13* inhibited adipocyte differentiation in intramuscular adipocytes. (**A**) *ACOT13* levels were reduced by the si-ACOT13. (**B**) Levels of *ACOX3*, *ATGL*, PPARD, and *FABP4* mRNAs decreased after transfection with si-ACOT13. (**C**) si-ACOT13 decreased the lipid droplet formation in intramuscular adipocytes by Oil Red O staining. (**D**) After transfection of si-ACOT13, triacylglycerol synthesis was decreased. * *p* < 0.05; ** *p* < 0.01.

Second, knockdown of *ACOT13* in chicken intramuscular preadipocytes. As we expected, the expression level of *ACOT13* and marker genes involved in fatty acid β-oxidation (*ACOX3* and *ATGL*) and adipogenesis (*PPARA*, *PPARD*, *CEBPA,* and *FABP4*) were significantly lower in ACOT13 knockdown group than that in the control group (Figure 7A,B). Furthermore, evidence from Oil Red O staining and intracellular triglyceride assay demonstrated that lipid droplet accumulation and intracellular triglyceride content significantly declined in the ACOT13 knockdown group (Figure 7C,D).

**Figure 8:**
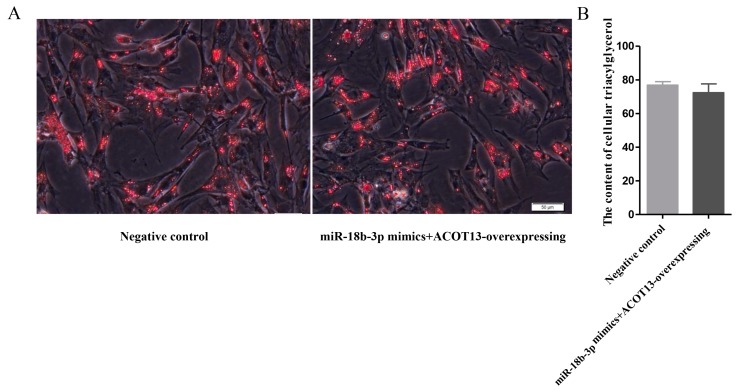
Rescue experiment of *ACOT13*, co-transfecting with miR-18b-3p mimics and ACOT13 overexpression. (**A**) Lipid droplet formation between groups. (**B**) Triacylglycerol content between groups.

Third, co-transfection of gga-miR-18b-3p mimicswith *ACOT13* overexpression plasmid was performed to further verify the effects of gga-miR-18b-3p and ACOT13 on preadipocyte differentiation. The treatment of negative control mimics together with the negative control plasmid was also conducted. The results showed that there was no significance in both lipid droplet accumulation and intracellular triglyceride concentration between the two treatments (Figure 8A,B).

Taken together, overexpression of *ACOT13* promoted preadipocyte differentiation, fatty acid β-oxidation, and adipogenesis. Conversely, knockdown of *ACOT13* would inhibit these processes. Moreover, the promoting effect of *ACOT13* on preadipocyte differentiation, fatty acid β-oxidation and adipogenesis can be eased by the inhibitory effect of gga-miR-18b-3p. All the results demonstrated that gga-miR-18b-3p targeting *ACOT13* inhibited intramuscular adipocytes differentiation.

## 4. Discussion

In recent years, understanding the mechanisms underlying the process of adipocyte differentiation has vastly expanded. Several potential miRNAs in our miRNA-seq data have been reported to be associated with cell differentiation, such as miR-221, miR-146, miR-30-5p, miR-301, and miR-148 [38]. Some of them played pivotal roles in the differentiation of adipocytes [39,40], which was consistent with our expected results, that we could obtain the differentially expressed miRNAs related to adipocyte differentiation.

KEGG pathway analysis was performed to investigate the biological interpretation of target genes for differentially expressed miRNAs. Three important pathways involved in fatty acid metabolism were excessively enriched, including the PPAR signaling pathway (gga03320), fatty acid metabolism (gga01212), and fatty acid degradation (gga00071). The PPAR signaling pathway is a very important intermediate metabolic pathway. Fatty acids are able to activate the expression of genes via PPARs [41]. PPARs are transcription factors that are ligand-activated, and they belong to the superfamily of nuclear hormone receptors. PPARα, PPARβ/δ, and PPARγ are included in PPARs. These three transcription factors act on their target genes and participate in lipid metabolism and adipocyte differentiation. Recent research found that PPARs had an influence on adipocyte differentiation [42,43] and fatty acid oxidation [44,45]. The marker gene, *FABP* [46], has been proven to have the same function in the PPARs signaling pathway. In the pathway of fatty acid metabolism and fatty acid degradation, *ACOX1* [47] is also involved in fatty acid oxidation, while *FASN* [48] and *ELOVL5* [49] are related to fatty acid biosynthesize.

Recent studies have found that miR-18b was considered as important marker of cell proliferation and cell adhesion. Murakami Y et al. found that the expression level of miR-18b was significantly associated with histological differentiation. Poorly differentiated hepatocellular carcinoma (HCC) presented high miR-18b and low TNRC6B (trinucleotide repeat containing 6B) expression levels. The mechanism underlying was that miR-18b inhibited HCC differentiation via targeting the TNRC6B gene [50]. Namløs HM et al. also reported that the expression of miR-18b in osteoblast and normal bone was identical with the differentiation level of osteosarcoma. From osteoblasts to osteosarcoma, then to bone, the degree of cell differentiation gradually increased. The higher the degree of cell differentiation, the lower the expression of miR-18b [51]. Both above studies suggested that miR-18b might function as a crucial factor in cell differentiation. Therefore, we concluded that miR-18b participated in the cell differentiation process. At the same time, miR-18b-3p was also a differential expressing miRNA between IMF-preadipocytes and IMF-adipocytes. We inferred that miR-18b-3p should be involved in the process of IMF preadipocytes. Meanwhile *ACOT13* was one of the differential genes in IMF-preadipocytes and IMF-adipocytes (unpublished data). Prediction of miRNA target genes suggested *ACOT13* was the target of gga-miR-18b-3p.

Acyl-CoA thioesterase 13 (*ACOT13*), a member of the acyl-CoA thioesterase (ACOT) gene family, catalyzed the key steps in fatty acid biosynthesis, bioluminescence, and non-ribosomal peptide synthesis [52]. *ACOT13* abundantly expressed in oxidative tissues (e.g., liver, adipose tissue) [53] and co-localized with mitochondria and long-chain fatty acyl-CoA substrates [54]. ACOT gene family could catalyze the hydrolysis of fatty acyl-CoAs to form free fatty acids (FFAs) and coenzyme A (CoASH). Indeed, the balance between FFAs and fatty acyl-CoAs within cells regulated fatty acid oxidation and controlled the synthesis of complex lipids [55,56]. Hung J Y et al. found that *ACOT11* and *ACOT13* knockdown-mediated growth inhibition could be rescued by the addition of fatty acids [57]. Nicholls previous studies have revealed that activated *ACOT13* at the mitochondrial membrane could result in increased fatty acid oxidation [58]. All these findings showed that *ACOT13* took part in lipid metabolism and fatty acid β-oxidation. In this study, results from overexpression and knockdown of the *ACOT13* gene demonstrated that *ACOT13* played an important role in fatty acid β-oxidation and adipogenesis, which were all consistent with previous studies. 

Zhang et al. reported that miR-140-5p affected IMF deposition in chicken with the association with meat quality [4]. However, gga-miR-140-5p was not the top miRNA in our present study. The differentially expressed miRNAs in Zhang’s study were detected between 20 and 55 weeks in Gushi chicken, so the difference might be not only caused by the different IMF deposition, but also by different stages between groups. As a short-chain non-coding small RNA, the expression of miRNAs in different tissues, and stages were different. In addition, miRNAs have coordinated biological functions by targeting genes, meanwhile, the target interactions were different in different tissues and stages [59,60]. Although miR-140-5p had potential effects on cell growth, migration, and death [61] in many species, such as pig [62], human, and chicken [63,64], many gene were involved in the process. Therefore, the direct effect between preadipocytes and adipocytes in our study might not be caused by miR-140-5p.

In our study, we identified a candidate miRNA, gga-miR-18b-3p, involved in the adipocyte differentiation based on the results of miRNA-seq. Bioinformatics analysis and functional assay suggested that gga-miR-18b-3p inhibited the differentiation of chicken intramuscular preadipocytes via targeting the *ACOT13* gene.

## 5. Conclusions

**Figure 9:**
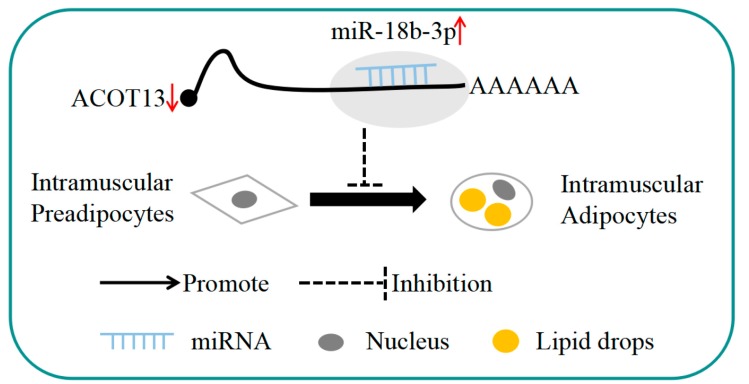
Proposed model of gga-miR-18b-3p regulation on intramuscular adipocyte differentiation.

In conclusion, our study identified that miRNAs were associated with chicken preadipocyte differentiation between IMF-preadipocytes and IMF-preadipocytes. This study demonstrated that gga-miR-18b-3p could inhibit the intramuscular adipocytes differentiation by negatively regulating *ACOT13* (Figure 9). These findings may provide fundamental basis for improving poultry meat quality, and it will also be helpful for a deeper understanding of chicken IMF deposition.

## Figures and Tables

**Table 1:** Primer sequences used for qPCR in this study.

Gene	Acc. #	Forward Primer Sequence 5’-3′	Reverse Primer Sequence 5′-3′
ACOT13	*XM_015282414.2*	CTTTGGAACAGCAGCACAGTT	TTACAGGAACCCTTCACTGCC
*β-actin*	*NM_205518.1*	CAGCCAGCCATGGATGATGA	ACCAACCATCACACCCTGAT
siRNA	*XM_015282414.2*	GCACGUUACACGGAGGUUUTTAAACCUCCGUGUAACGUGCTT	
*ACOX1*	*NM_001006205.1*	AAGGAGATCGAGGCCTTAGTG	GCCGTCCACGATGAACAAAG
*ACOX3*	*XM_420814.6*	AAAGAAGACAGTGGCAACCG	TCACCATCGAGAAACACTGC
*ATGL*	*NM_001113291.1*	CACTGCCATGATGGTCCCCTA	CCACAAGGAGATGCTGAAGAA
*PPARA*	*XM_015289937.2*	AGTAAGCTCTCAGAAACTTTGTTG	GTCATTTCACTTCACGCAGCA
*PPARD*	*XM_015298893.2*	GCAAGCCCTTCAACGAGATCA	GGGACGATCTCCACACAGG
*CEBPA*	*NM_001031459.1*	TTCTACGAGGTCGATTCCCG	AGCCTCTCTGTAGCCGTAG
*FABP4*	*NM_204290.1*	ATGTGCGACCAGTTTGT	TCACCATTGATGCTGATAG
PPARG	*XM_015292931.2*	GTGCAATCAAAATGGAGCC	CTTACAACCTTCACATGCAT
ACOT13(CDS)	*XM_015282414.2*	CCCAAGCTTGCCACCATGGGCAGCATGCGCTTCAC	CCGGAATTCCTGTCCTATGTACTTTGTAT
WT-ACOT13	*ENSGALT00000086884.1*	CCGCTCGAGAAGCAACTCACCCTTCAGGC	ATTTGCGGCCGCAGCTTGCTTCACACTTCCCAT
MUT-ACOT13	*ENSGALT00000086884.1*	CTTACTAATTGTTCCGTAGGTCCCGTCAGAAGGGTTCCTGTAAGCAAC	GTTGCTTACAGGAACCCTTCTGACGGGACCTACGGAACAATTAGTAAG

**Table 2:** The eight significantly-enriched pathways related to lipid metabolism.

Pathway ID	Pathway Term	Target Gene List
gga00062	Fatty acid elongation	ELOVL5, HACD3, HADHA, ACAA2, ELOVL4, HACD1, ECHS1, ELOVL1, HSD17B12, MECR, PPT1, ACOT7, HACD2, ELOVL6
gga00020	Citrate cycle	DLD, IDH3B, IDH3A, SDHA, ACLY, SUCLG2, SUCLG1, ACO2, MDH1, IDH1, ACO1, OGDH, SDHB, IDH3G, IDH2, CS
gga01212	Fatty acid metabolism	ELOVL5, HACD3, ACADS, EHHADH, HADHA, ACAA2, SCD5, FASN, ACSBG2, ACOX1, HACD1, ACSBG1, ECHS1, SCD, HSD17B12, MECR, PPT1, ACSL1, PECR, CPT2, HACD2,
		ACADSB, ACADL, ELOVL6
gga00071	Fatty acid degradation	ACADS, EHHADH, HADHA, ACAA2, ACSBG2, ACOX1, ECI1, ACSBG1, ECHS1, ACSL1, CPT2, ALDH7A1, ECI2, ACADSB, ACADL
gga04010	MAPK (mitogen-activated protein kinase) signaling pathway	RELA, CHUK, TAOK3, BDNF, EGFR, TNFSF6, PAK1, ChALK5, TAB1, TGFB3, ARR3, RAC2, MAP2K1, NF1, FOS, MAP3K13, PRKCA, CRKL, DUSP6, SRF, CACNG5, GADD45A, FGFR4, MAP3K14, HSPA2, NRAS, cRac1B, PDGF-A,CACNA1D, CASP3, CDC42, MAPK10, STK4, PTPN7, TGFBR2, RPS6KA5, FGF1, DUSP4, GNG12, DUSP7, MAP3K1, NR4A1, HSPA8, LAMTOR3, PPP3CB, TAOK1, MRAS, CACNA1S, MAP2K5, MAPK11, PPP3R1, NFATC3, DUSP10, CACNA1G, RPS6KA, DUSP3, cRac1A, FLNB, CACNA2D1, H-RAS, GADD45B, PPM1B, MAPKAPK3, CACNA1B, TNFRSF1A, BRAF, FGFR1, NFKB2, FGFR3, MAP3K5, CACNG4, FGF8, MAPK9, CACNG3, MAP2K3
gga04150	mTOR (mammalian target of rapamycin) signaling pathway	CHUK, ATP6V1G1, PDPK1, SLC7A5, STRADA, RNF152, PIK3R5, FZD5, MAP2K1, CAB39L, RPS6KB1, WDR24, SKP2,PRKCA, ATP6V1G3, NPRL2, WNT6, STK11, FZD6, FZD9, SLC38A9, ATP6V1B2, RPS6, FZD4, NRAS, WDR59, MLST8, Wnt8c, RRAGC, PIK3CB, PIK3CD, RRAGD, LAMTOR3, FZD3, ATP6V1A, RPTOR, ULK3, ATP6V1D, CLIP1, WNT11, RPS6KA, PRKAA1,HRAS, EIF4E2, PRKAA2, TNFRSF1A, BRAF, RHOA, TBC1D
gga00061	Fatty acid biosynthesis	FASN, ACSBG2, ACSBG1, ACSL1, ACOT13
gga03320	PPAR (peroxisome proliferator-activated receptors) signaling pathway	FABP5, PDPK1, EHHADH, FABP3, LXR, PLIN2, SCD5, ACSBG2, LPL, ACOX1, SLC27A2, ACSBG1, SCD, ACSL1, CPT2, CYP8B1, PLIN1, ACADL

## Supplementary Materials

The following are available online at https://www.mdpi.com/2073-4409/8/6/556/s1. Table S1: Identification of differentially expressed miRNAs; Table S2: Gene ontology (GO) terms enrichment analysis.

## Accession Numbers

All the Illumina miRNA-seq data sets supporting the results of this article have been submitted to the National Center for Biotechnology Information (NCBI) Gene Expression Omnibus (GEO). Accession numbers are SAMN08325464, SAMN08325465, SAMN08325466, SAMN08325467.

## Abbreviations

ACOX1	acyl coenzyme A oxidase 1: palmitoyl
ACOX3	acyl coenzyme A oxidase 3, palmitoyl
ATGL	adipose triglyceride lipase
PPARA	peroxisome proliferator-activated receptor α
PPARD	peroxisome proliferator-activated receptor β
CEBPA	CCAAT-enhancer binding protein-alpha
FABP4	fatty acid binding protein 4
ACOT13	acyl-CoA thioesterase 13
IMF	Intramuscular fat
PBS	phosphate buffered saline
TG	triacylglycerol
GO	gene ontology
KEGG	Kyoto encyclopedia of genes and genomes
